# Body Mass Index and Sex Affect Diverse Microbial Niches within the Gut

**DOI:** 10.3389/fmicb.2018.00213

**Published:** 2018-02-14

**Authors:** Francesca Borgo, Stefania Garbossa, Alessandra Riva, Marco Severgnini, Carmelo Luigiano, Albero Benetti, Antonio E. Pontiroli, Giulia Morace, Elisa Borghi

**Affiliations:** ^1^Department of Health Sciences, Università degli Studi di Milano, Milan, Italy; ^2^ASST Santi Paolo e Carlo Hospital, Milan, Italy; ^3^Division of Microbial Ecology, Department of Microbiology and Ecosystem Science, Research Network “Chemistry meets Microbiology”, University of Vienna, Vienna, Austria; ^4^Institute of Biomedical Technologies, National Research Council, Segrate, Italy

**Keywords:** luminal microbiota, mucosa-associated microbiota, body mass index, sex, indicative species

## Abstract

Gut microbiota is considered a separate organ with endocrine capabilities, actively contributing to tissue homeostasis. It consists of at least two separate microbial populations, the lumen-associated (LAM) and the mucosa-associated microbiota (MAM). In the present study, we compared LAM and MAM, by collecting stools and sigmoid brush samples of forty adults without large-bowel symptoms, and through a 16S rRNA gene next-generation sequencing (NGS) approach. MAM sample analysis revealed enrichment in aerotolerant *Proteobacteria*, probably selected by a gradient of oxygen that decreases from tissue to lumen, and in *Streptococcus* and *Clostridium* spp., highly fermenting bacteria. On the other hand, LAM microbiota showed an increased abundance in *Bacteroides, Prevotella*, and *Oscillospira*, genera able to digest and to degrade biopolymers in the large intestine. Predicted metagenomic analysis showed LAM to be enriched in genes encoding enzymes mostly involved in energy extraction from carbohydrates and lipids, whereas MAM in amino acid and vitamin metabolism. Moreover, LAM and MAM communities seemed to be influenced by different host factors, such as diet and sex. LAM is affected by body mass index (BMI) status. Indeed, BMI negatively correlates with *Faecalibacterium prausnitzii* and *Flavonifractor plautii* abundance, putative biomarkers of healthy status. In contrast, MAM microbial population showed a significant grouping according to sex. Female MAM was enriched in *Actinobacteria* (with an increased trend of the genus *Bifidobacterium*), and a significant depletion in *Veillonellaceae*. Interestingly, we found the species *Gemmiger formicilis* to be associated with male and *Bifidobacterium adolescentis*, with female MAM samples. In conclusion, our results suggest that gut harbors microbial niches that differ in both composition and host factor susceptibility, and their richness and diversity may be overlooked evaluating only fecal samples.

## Introduction

The gastrointestinal tract is the major reservoir for a complex microbial community, called gut microbiota (Human Microbiome Project Consortium, 2012). The gut microbiota can be considered a separate endocrine organ that participates, through a continuous molecular crosstalk with the host, in the maintenance of energy homeostasis and in the stimulation of host immune system (Clarke et al., 2014). Studies conducted on humans and germ-free mice have shown the functional role of the gut microbiota in promoting health status (Grover and Kashyap, 2014; Marchesi et al., 2015). Indeed, changes in gut microbiota composition have been associated to various human diseases such as inflammatory bowel diseases (IBD) (Ferreira et al., 2014), irritable bowel syndrome (IBS) (Lee and Lee, 2014), metabolic diseases (obesity and diabetes) (Turnbaugh and Gordon, 2009; Flint et al., 2012), allergic diseases (Ismail et al., 2012) and neurological disorders (Borre et al., 2014; Borgo et al., 2017; Borghi et al., 2017).

From a bacterial point of view, the gut microbiota consists of two separate microbial populations, the lumen-associated (LAM) and the mucosa-associated microbiota (MAM) (Van den Abbeele et al., 2011). Luminal microbiota is influenced by changes in diet and luminal content, whereas MAM is considered to be relatively stable in individuals throughout their life (Li et al., 2008). The increased stability is, partially, dependent on the ability of these microbes to attach to the host mucosa and to settle on a niche through biofilm formation and inhibiting other microbes’ growth. This intimate association influences host physiology in health and the development of disease (Backhed et al., 2004; Heinsen et al., 2015; Shobar et al., 2016). A recent study by Haro et al. (2016) on human fecal microbiota highlighted differences between men and women in the luminal microbial population. In addition to that, total body fat content also seems to influence microbiota composition (Mestdagh et al., 2012), widening sex-related differences.

The majority of literature studies encompass analysis of the luminal microbiota (stool samples), assuming it as an indicator for the entire gut microbial population. To our knowledge, no study focused on the influence of body mass index (BMI) and sex on MAM, whereas some data reported its contribution in chronic constipation (Parthasarathy et al., 2016), IBS (Ouwehand et al., 2004) and colorectal cancer (Chen et al., 2012).

Thus, the aims of our study were: (i) to compare mucosa-associated to fecal microbiota using high throughput next-generation sequencing (NGS) and (ii) to evaluate possible host factors that could influence the microbial biodiversity in the two niches.

## Materials and Methods

### Ethics Statement

This study was carried out in accordance with the recommendations of Comitato Etico Interaziendale Milano Area A, protocol number 173/ST/2014. All subjects gave written informed consent in accordance with the Declaration of Helsinki.

### Subjects and Sampling

Participants were enrolled from the Medical Department of the ASST Santi Paolo e Carlo of Milan, Italy. All recruited subjects underwent a screening colonoscopy for preventive purpose (in the context of the Italian prevention campaign for early diagnosis of colon cancer). Exclusions criteria were: type 2 diabetes; antibiotic therapy, probiotic or prebiotic supplementation in the previous 6 months; oncological diseases; intestinal chronic inflammatory diseases; alcohol consumption greater than 20 g/day; liver diseases (including infectious hepatitis, α-1-antitrypsin deficiency, Wilson disease); celiac disease and polycystic ovary syndrome. All subjects performed, before colonoscopy, the same bowel cleansing (MOVIPREP, Norgine, Milan, Italy, 2 doses: 1 L the evening before and 1 L the same morning of the colonoscopy) according to manufacturer’s instructions. Samples from sigmoid colon brushing were collected during routinely colonoscopy performed at the Endoscopy Unit of ASST Santi Paolo e Carlo of Milan by means of a Microbiological Protected Specimen Brush (AORTA s.r.l., Milan, Italy). Sterile single-use brushes have a distal plug at the tip that seals the brush within the sheath during introduction and retraction through the colon. Gently brushing allowed superficial mucus gel layer collection without residual fluid contamination. After procedure, the brush was sealed, put in a 15 ml tube and stored at -80°C until further processing.

Stool samples were collected 3 weeks after the colonoscopy and stored at -80°C. Each subject underwent a venipuncture to perform blood tests [blood fasting glucose, insulin, glycated hemoglobin, total cholesterol, HDL cholesterol, LDL cholesterol, triglycerides, Alanine amino transferase (ALT), Aspartate amino transferase (AST), Gamma glutamil-transpeptidase (γGT), Alcaline phosphatase (ALP), total protein, serum protein electrophoresis, creatinine, urea, electrolytes, C reactive protein (CRP), thyroid stimulating hormone (TSH), complete cell count]. All biochemical parameters were analyzed using routine laboratory methods.

Anthropometric data (weight, height, and body mass index-BMI) were recorded at the time of enrolment.

### Food Habits, Eating Behavior, and Physical Activity Assessment

Before fecal sample collection, all subjects filled out a 3-day food diary to evaluate eating habits. Dieticians calculated the energy and nutrient intakes according to the *Italian aliments composition database for epidemiological studies* (BDA-IEO^1^).

Lifestyle and physical activity were defined by a self-administered questionnaire as reported by Turconi et al. (2008).

### Bacterial DNA Extraction and 16S rRNA Gene Sequencing

Genomic DNA was extracted from brush samples by using the QIAamp DNA Microbiome Kit (QIAGEN, Hilden, Germany). Collected samples were dislodged from brushes by vigorous agitation after adding 1 ml of phosphate buffered saline (PBS) to 15 mL tube. Subsequent steps were performed according to manufacturer’s recommendations. Briefly, depletion of host cells was performed by adding lysis buffer and benzonase to samples. Bacterial cells lysis was then achieved using Pathogen Lysis Tubes (containing large beads) and a lysis buffer in the TissueLyser LT instrument (QIAGEN). Lysates were transferred to QIAamp UCP Mini Columns and bound DNA was eluted in 50 μl of buffer. Bacterial genomic DNA in stool samples was extracted by using the Spin stool DNA kit (Stratec Molecular, Berlin, Germany), according to the manufacturer’s instructions. Briefly, after homogenizing fecal samples in the lysis buffer for inactivating DNases, Zirconia Beads II were added for a complete lysis of bacterial cells by using TissueLyser LT. Bacterial lysates were then mixed with InviAdsorb reagent, a step designed to remove PCR inhibitors. Bacterial DNA, bound to the membrane RTA Spin Filter, was eluted in 100 μl of buffer. Library preparation and 16S rRNA NGS were performed as previously reported, using the Illumina MiSeq platform (Borgo et al., 2016; Borghi et al., 2017).

Next-generation sequencing raw reads were processed merging read pairs by using PandaSeq software (“PAired-eND Assembler for DNA sequences”) (Masella et al., 2012) and quality-filtered using the “split_libraries_fastq.py” utility of the QIIME suite (Caporaso et al., 2010), filtering out sequences having more than 25% nucleotides with a phred score of 3 or less. Quality-filtered reads were analyzed with the standard QIIME pipeline. Sequences were grouped into OTUs (operational taxonomic units) by using UCLUST (Edgar, 2010) with 97% similarity threshold and taxonomically classified against the 13.8 release of the Greengenes bacterial 16S rRNA database^2^ by RDP classifier (Wang et al., 2007) at 50% confidence. Singletons (i.e., OTUs having only 1 supporting read along the whole 80-samples dataset) were considered possible chimeras and thus discarded. Sequencing libraries for luminal microbiota were subsampled to at most 100,000 reads per sample.

Raw sequence data have been deposited in NCBI Short-Reads Archive (SRA) under BioProject PRJNA401981.

### Statistical Analysis

Statistical analysis was performed using the statistical software Matlab (Natick, MA, United States) and R platform^3^. When performing analysis on LAM and MAM population separately, obvious outlier samples were removed from the dataset. This included five luminal samples (F3, F12, F15, F21, and F25) and three mucosal samples (M10, M17, and M29), all characterized by a very low biodiversity, with very few bacterial groups accounting for the majority of their composition. Sample biodiversity (i.e., alpha diversity evaluation) was estimated according to different microbial diversity metrics (i.e., chao1, Shannon index, observed species and Faith’s phylogenetic distance). Inter-sample diversity (i.e., beta-diversity) was calculated using both weighted and unweighted Unifrac metrics (Lozupone and Knight, 2005) and Principal Coordinates Analysis (PCoA). Data separation was tested with a permutation test with pseudo *F*-ratios (function “adonis”) and the significant clustering of groups was evaluated with analysis of similarities (ANOSIM) in the “vegan” package (Oksanen et al., 2013). Indicator species analysis was performed using the “indicspecies” package (De Cáceres and Legendre, 2009). For relative abundance analysis, a Mann–Whitney *U*-test was used; a *p*-value < 0.05 was chosen as threshold for statistical significance.

The relationships between differential OTUs were evaluated by Spearman’s rank correlation. Sequences alignment were performed by using the basic local alignment tool (BLAST) program (Altschul et al., 1997), from the National Center For Biotechnology Information BLAST website^4^, against the “nr” database with default settings.

Phylogenetic Investigation of Communities by Reconstruction of Unobserved States (PICRUSt) 1.0.0 (Langille et al., 2013) was applied to predict metagenome function from the 16S rRNA gene data; Bray–Curtis distances were used to determine similarity of samples based on metagenomic composition. Differences in the taxa and predicted molecular functions were analyzed by the linear discriminant analysis (LDA) effect size (LEfSe) (Segata et al., 2011) with default settings (Alpha value for the factorial Kruskal–Wallis test among classes = 0.05; Threshold on the logarithmic LDA score for discriminative features = 2.0)^5^.

## Results

### Cohort Description

Fecal and brush samples were collected from 40 subjects between January 2015 and January 2016. All participants were Caucasian and living in Northern Italy. Bowel preparation, assessed during colonoscopy by clinician, was considered suitable in all subjects. Enrolled subjects did not show any pathological trait during colonoscopy examination. Participants, sex- and age-matched, were split in two groups based on BMI score: 20 subjects with BMI < 25 (mean ± SD; 22.8 ± 1.8), classified as normal weight (NW), and 20 with BMI > 30 (35.8 ± 8.3), classified as obese (O). **Table 1** lists the characteristics of the study population, and summarizes significant biochemical parameters. Diet analysis did not show significant differences between the two groups. Indeed, obese subjects declared correct eating habits (65%) and a sedentary lifestyle (80%).

**Table 1:** Anthropometric data and significant biochemical parameters of the study population.

	NW (*n* = 20)		O (*n* = 20)		Reference range^§^
			
	Male	Female	Male	Female	
Age (year)	48.7 ± 10.2	51.7 ± 8.3	53.8 ± 7.7	51.3 ± 6.7	
Weight (kg)^∗∗∗^	71.2 ± 5.1	61.7 ± 6.6	100.6 ± 16.6	92.9 ± 23.6	
Height (cm)	176.2 ± 4.8	164.7 ± 5.9	173.3 ± 4.9	156.5 ± 5.3	
BMI (kg/m^2^)^∗∗∗^	22.9 ± 1.7	22.7 ± 1.6	34.6 ± 4.4	35.6 ± 5.1	
WC (cm)^∗∗∗^	83.1 ± 2.4	82.9 ± 3.2	112.1 ± 8.5	109.3 ± 9.8	
Insulin (μU/ml)^∗∗^	6.1 ± 3.0	5.5 ± 2.3	15.6 ± 7.3	14.1 ± 3.4	0–25
HOMA-ir^∗∗^	1.4 ± 0.8	0.9 ± 0.4	3.4 ± 1.8	3.2 ± 0.9	0.22–2.5
HDL (mg/dL)^∗∗∗^	59.7 ± 19.6	78.5 ± 20.7	43.4 ± 10.9	50.3 ± 7.7	>42
LDL (mg/dL)^∗∗^	114.4 ± 26.2	99.6 ± 24.2	134.0 ± 33.1	151.8 ± 42.4	<100
Triglycerides (mg/dL)^∗∗^	105.3 ± 52.2	77 ± 27.4	168.9 ± 63.6	127.8 ± 60.0	<150
WBC count (10^3^/μL)^∗^	6.4 ± 1.3	5.5 ± 0.5	6.6 ± 0.9	7.2 ± 1.7	3.6–9.2


**Data are expressed as mean ± standard deviation. O, obese; NW, normal weight; BMI, body mass index; WC, waist circumference; HOMA-ir, Insulin resistance index; HDL, high density lipoprotein cholesterol; LDL, low density lipoprotein cholesterol; WBC, white blood cell. ^§^Reference range for biochemical parameters. In the first column, statistical significant values (^∗^p ≤ 0.05, ^∗∗^p < 0.001, ^∗∗∗^p < 0.0001) refer to O vs. NW comparison.**

### Lumen and Mucosa Harbor Different Microbial Communities

**FIGURE 1:**
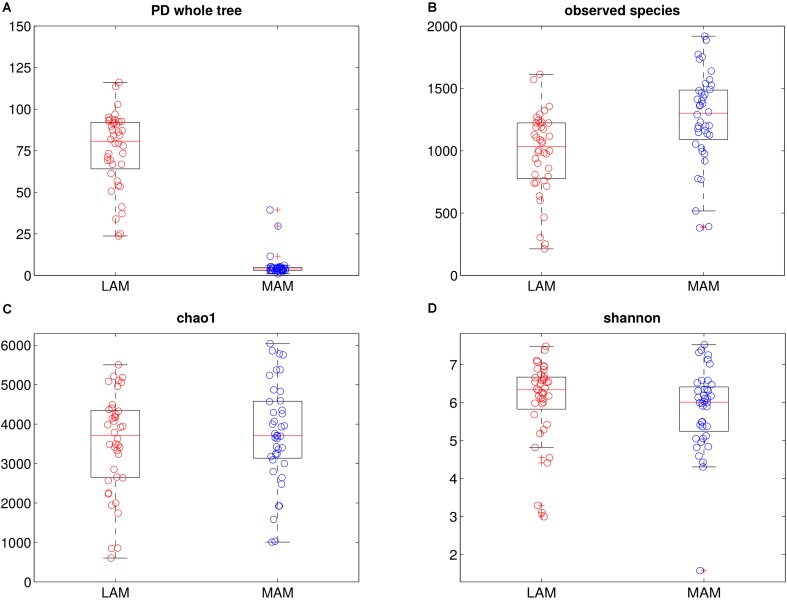
Intra-samples diversity of LAM and MAM microbiota. Boxplots of end-point α-diversity of LAM and MAM samples according to Faith’s phylogenetic diversity **(A)**, observed species **(B)**, chao1 **(C)** and Shannon **(D)** indexes. MAM samples (blue) have significantly lower values than LAM (*p* = 0.001, red) for Faith’s phylogenetic diversity metric, whereas, show a significantly higher number of observed species (*p* = 0.001).

16S rRNA gene sequencing data were processed to evaluate bacterial communities (**Figure 1**) inhabiting the two gut niches, lumen- (LAM) and mucosa-associated (MAM).

Mucosa-associated microbiota was sampled by sigmoid brush, a low invasive technique that has been previously demonstrated to be suitable in providing a good mucosal coverage and data comparable with biopsy samples (Huse et al., 2014). To minimize procedural differences, all enrolled subjects used, before colonoscopy, a colon cleansing preparation that was shown to not affect intestinal microbiota composition (Jalanka et al., 2015). This observation suggests that our results from brush samples are representative of MAM microbiota.

α-diversity (i.e., diversity within samples) was measured by OTU-based and phylogenetic tree-based (Chao1, observed species, Shannon and PD whole tree indexes, **Figure 1**) methods. Overall, MAM samples showed a higher diversity in terms of observed species (*p* = 0.001), whereas PD whole tree metrics showed a significant (*p* = 0.001) increase of phylogenetic distances among LAM populations.

**FIGURE 2:**
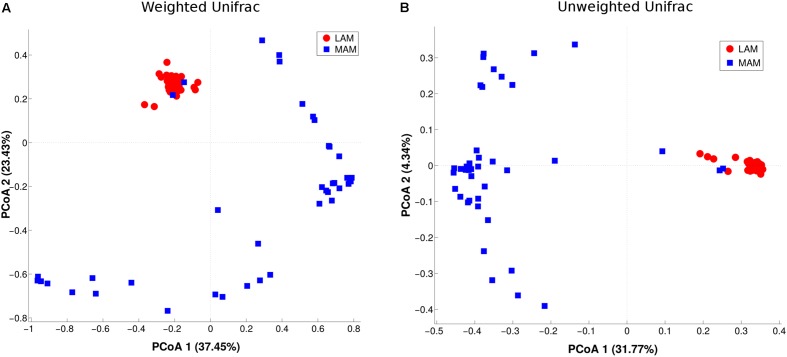
Inter-samples diversity of LAM and MAM microbiota. Principal Coordinate Analysis (PCoA, beta-diversity) plot according to weighted **(A)** and unweighted **(B)** Unifrac distances. The first two components of the variance are represented plotting LAM (red) vs. MAM (blue) samples. The two groups are significantly separated (*p* = 0.01).

β-diversity (i.e., diversity between the two populations) was evaluated by PCoA analysis (**Figure 2**). The test highlighted a statistically significant separation between LAM and MAM groups according to both unweighted (*p* = 0.01) and weighted Unifrac distances (*p* = 0.01).

On the other hand, taking together LAM and MAM samples, no separation (*p* > 0.05) was obtained in the comparison between either obese and NW subjects or between males and females (Supplementary Figure S1).

The evaluation of Unifrac distances of paired LAM and MAM samples from the same patient showed no statistical difference (*p* > 0.05) between intra- and inter-patient distances (data not shown).

**FIGURE 3:**
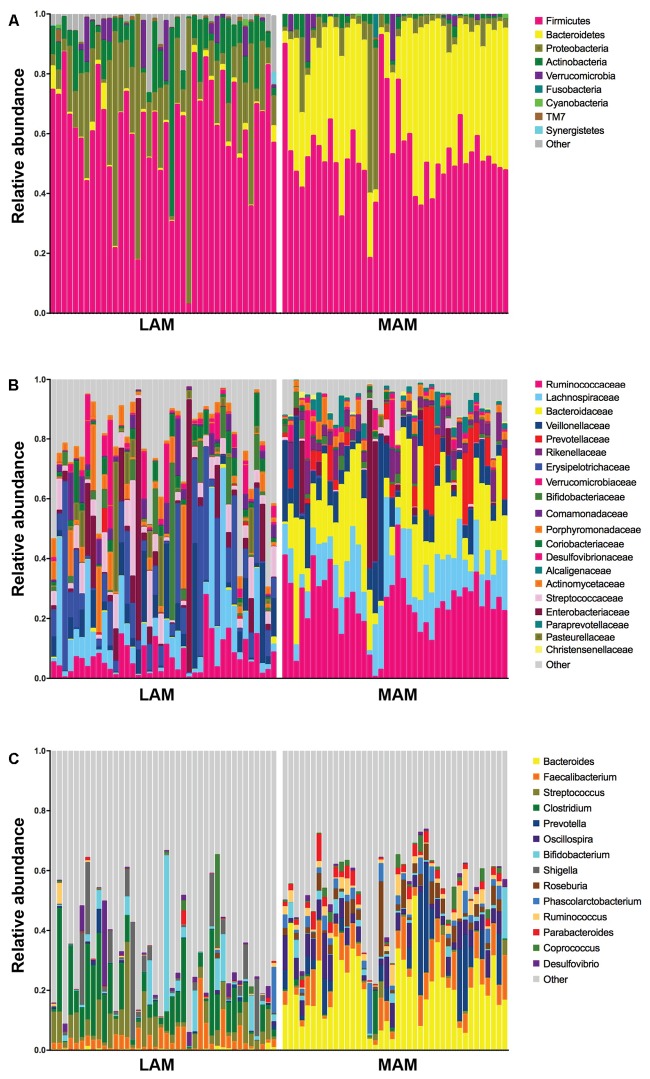
Relative abundances in lumen-associated and mucosa-associated microbiota. Histograms of relative abundances at phylum **(A)**, family **(B)**, and genus **(C)** level for all subjects (*n* = 40). MAM group refers to brush samples, whereas LAM group to fecal samples.

**Figure 3** shows relative abundances, at various taxonomic levels, of the most represented bacteria in each studied sample. The most abundant and significantly different taxa are reported in **Table 2**.

**Table 2:** Most abundant and significantly different taxa in MAM and LAM samples.

	TAXA	MAM	LAM
Phylum	*Firmicutes*	61.3 ± 18.9	53.1 ± 14.1
	*Proteobacteria*	20.1 ± 21.6***	6.3 ± 11.4
	*Actinobacteria*	10.1 ± 10.0***	1.7 ± 1.7
	*Bacteroidetes*	1.4 ± 1.9	37.1 ± 14.1***
	*Verrucomicrobia*	2.4 ± 4.9	1.3 ± 3.6
Family	*Erysipelotrichaceae*	13.7 ± 15.2***	2.7 ± 2.0
	*Lachnospiraceae*	10.3 ± 13.3	13.6 ± 6.5**
	*Ruminococcaceae*	7.1 ± 5.8	25.1 ± 10.3***
	*Bacteroidaceae*	0.2 ± 0.4	20.2 ± 12.1***
Genus	*Bacteroides*	0.3 ± 0.5	20.2 ± 12.0***
	*Prevotella*	0.3 ± 1.3	5.5 ± 9.3*
	*Oscillospira*	0.2 ± 0.3	5.2 ± 3.4***
	*Streptococcus*	6.0 ± 4.5***	0.5 ± 1.0
	*Clostridium*	5.7 ± 3.2***	0.6 ± 1.3
	*Enterobacter*	3.8 ± 2.5***	0.9 ± 0.1


**Data are expressed as mean ± standard deviation. MAM, mucosa-associated microbiota; LAM, lumen associated microbiota. Statistical significant values (^∗^p ≤ 0.05, ^∗∗^p < 0.001, ^∗∗∗^p < 0.0001) are reported highlighting the group with greater abundance.**

Mucosa-associated microbiota samples are significantly enriched in the phyla *Proteobacteria* and *Actinobacteria* and highly depleted in *Bacteroidetes* compared with LAM samples. At family level, MAM showed increased presence of *Erysipelotrichaceae* and marked decrease of *Ruminococcaceae* and *Bacteroidaceae*. Significantly different genera included: *Bacteroides*, *Prevotella*, and *Oscillospira* (increased in LAM microbiota) and *Streptococcus*, *Clostridium*, and *Enterobacter* (increased in MAM microbiota).

To gain insight into the molecular functions of bacterial microbiota, we used PICRUSt to predict the metagenomic contribution of the communities observed by imputing the available annotated genes within a known sequences database, the Kyoto Encyclopaedia of Genes and Genomes (KEGG).

**FIGURE 4:**
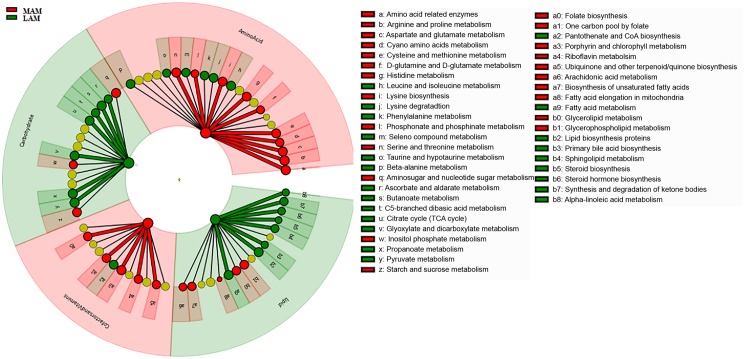
Predicted metabolic pathways enriched in LAM and MAM. Kyoto Encyclopedia of Genes and Genomes (KEGG) pathways differentially abundant in lumen- (LAM, red) and mucosa-associated (MAM, green) gut microbiota. Brightness is proportional to enzymes abundance. Cladogram represents the KEGG BRITE functional hierarchy: the outermost circles represent very broad functional categories, and innermost specific metabolic pathways.

PICRUSt analysis suggested significant differences (**Figure 4**) in several metabolic pathways. In particular, LAM showed an enrichment in genes encoding enzymes for carbohydrate and lipid metabolisms, whereas those involved in the amino acids and cofactors and vitamins pathway were increased in MAM samples.

### Microbial Differences in Lumen-Associated Microbiota According to BMI Groups

**FIGURE 5:**
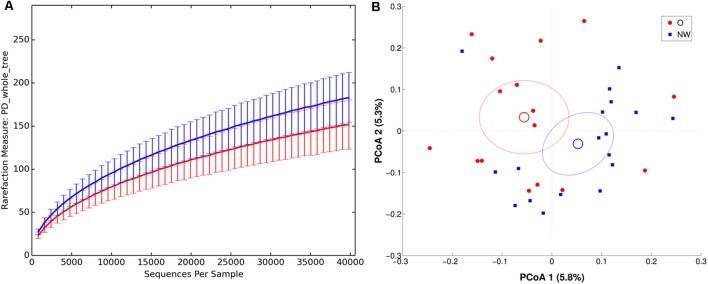
α and β diversity in LAM samples. NW samples are in blue, whereas O are in red. **(A)** α-diversity plot of LAM microbiota according to PD whole tree metric. Differences are statistically significant (*p* < 0.01). **(B)** PCoA according to unweighted Unifrac distance, showing a significant (*p* = 0.017) separation between the two groups.

Richness metrics of LAM communities correlated with BMI (all metrics *p* < 0.01) (**Figure 5A**), with a significant reduction of α-diversity in obese compared to NW subjects.

In agreement, both ANOSIM analysis, revealing a significant clustering of samples according to BMI at every taxonomic level (*p* < 0.05), and β-diversity analysis, showing a significant separation in LAM samples between the centroids of NW and O (unweighted Unifrac, *p* = 0.017; weighted Unifrac, *p* = 0.045) (**Figure 5**), confirmed this data.

*Oscillospira* genus (relative abundance, O: 4.9 ± 3.7, NW: 6.9 ± 2.3, *p* < 0.05) was significantly decreased in obese fecal samples. On the other hand, *Veillonellaceae* (O: 9.0 ± 4.8, NW: 4.2 ± 2.0, *p* < 0.005) and *Dialister* spp. (O: 3.6 ± 4.1, NW: 0.8 ± 1.4, *p* < 0.05) were significantly higher in O compared to NW subjects. A comprehensive table of relative abundances in LAM microbiota at every taxonomic level is provided in Supplementary Table S1.

Indicator species analysis (Spearman rank correlation analysis) revealed OTU 14127 (best BLAST hit: *Flavonifractor plautii*, Accession Number: NR_029356, with 98% seq. similarity over 420 bp, *p* = 0.04) and OTU 545477 (best BLAST hit: *Faecalibacterium prausnitzii* ATCC 27768, Accession Number: NR_028961, with 99% seq. similarity over 420 bp, *p* = 0.05) to negatively correlate with BMI.

Multivariate analysis did not reveal any correlation between the studied biochemical parameters and luminal associated microbiota composition.

### Microbial Differences in Mucosa-Associated Microbiota According to Sex and BMI

**FIGURE 6:**
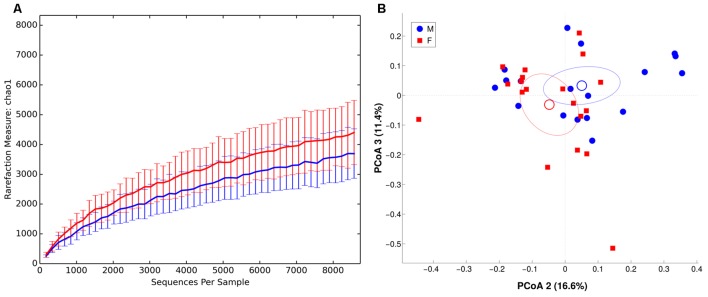
α and β diversity in MAM microbiota. Females are in red, whereas males are in blue. **(A)** α-diversity plot of MAM microbiota for chao1 metric. Differences are statistically significant (*p* < 0.05). **(B)** PCoA according to unweighted Unifrac distance, showing a significant (*p* = 0.049) separation between the two groups.

We observed males and females to be characterized by a different microbiota community composition at mucosal level (**Figure 6**, unweighted Unifrac distance, *p* = 0.049). On contrary, no significant differences were found between BMI groups. The intra-individual dissimilarity (α-diversity) in MAM microbiota composition showed a reduction of Chao1, observed species and Shannon metric (*p* < 0.05) in male subjects.

Female MAM microbiota was significantly enriched in *Actinobacteria* (M, male: 6.5 ± 4.7, F, female: 13.1 ± 14.2, *p* < 0.05) and *Lactobacillales* (M: 4.6 ± 3.6, F: 8.8 ± 7.6, *p* < 0.05). At lower taxonomic level, we observed a trend toward increased *Bifidobacterium* spp. (M: 1.0 ± 1.3, F: 6.2 ± 12.5) and *Streptococcaceae* (M: 4.1 ± 3.4, F: 8.2 ± 7.5), whereas *Veillonellaceae* (M: 10.6 ± 12.5, F: 3.5 ± 4.1, *p* < 0.05) and *unclassified Clostridia* (M: 1.2 ± 2.2, F: 0.3 ± 0.3, *p* < 0.05) were depleted. A comprehensive table of relative abundances in MAM microbiota, at every taxonomic level, is provided in Supplementary Table S2.

Indicator species analysis revealed OTU 351494 (Best BLAST Hit: *Bifidobacterium adolescentis* ATCC 15703, Accession Number: NR_074802, with 98% seq. similarity over 420 bp, *p* = 0.02) to be associated with females and OTU 228752 (Best BLAST Hit: *Phascolarctobacterium faecium*, Accession Number: NR_026111, with 97% seq. similarity over 422 bp, *p* = 0.03) and OTU 554378 (Best Blast Hit: *Gemmiger formicilis*, Accession Number: NR_104846, with 99% seq. similarity over 420 bp, *p* = 0.01) with males.

As for LAM, multivariate analysis did not reveal any correlation between the studied biochemical parameters and mucosa-microbiota composition.

## Discussion

In the present study, we investigated and compared the luminal and the MAM of 40 adult subjects without large-bowel symptoms. 16S rRNA sequencing revealed that the two sampled microbial communities, LAM and MAM, are influenced by different host factors: LAM, strongly diet-shaped (Zoetendal and de Vos, 2014; Xu and Knight, 2015; Singh et al., 2017), is associated to subjects’ BMI, whereas sex influences the composition of MAM, a community less susceptible to diet. This finding confirms previous studies describing a different microbial composition in luminal and mucosal microbiota (Ouwehand et al., 2004; Carroll et al., 2010; Codling et al., 2010; Parthasarathy et al., 2016) that highlighted significant changes in richness, evenness and relative abundances between fecal and mucosal samples.

Based on the nature of collected samples and in order to optimize microbial DNA recovery, we used two different DNA extraction commercial kits. Indeed, for fecal samples, a method encompassing a specific step to remove fecal PCR inhibitors was chosen. For mucosal brushes, we preferred a commercial kit with a step for host DNA depletion, specifically designed for samples characterized by low microbial DNA and high host DNA concentration. This strategy has already been used by other authors studying MAM and LAM communities (Carroll et al., 2010; Chen et al., 2012). Even authors using two different extraction methods for luminal and mucosal samples (Carroll et al., 2011; Burrough et al., 2017), concluded that these anatomical sites are two different microbial niches.

Our data suggest MAM to be characterized by a higher number of phylogenetically closer species than LAM samples. This profound difference is supported also by the lack of significant correlation between the LAM and MAM profiles of the same subjects. The most abundant phyla, in both MAM and LAM samples, were *Firmicutes*, *Bacteroidetes, Proteobacteria*, and *Actinobacteria*, although in different proportions. The two niches were characterized by different abundance in *Proteobacteria*, more represented in MAM samples. A diverse oxygen concentration could explain this phenomenon, as host cells generate a gradient of oxygen that decreases from tissue to lumen. This selective pressure allows aerotolerant bacteria, such as *Proteobacteria*, to successfully colonize the mucosal niche (Donaldson et al., 2015). *Streptococcus* and *Clostridium*, belonging to the *Firmicutes* phylum, were also significantly higher in MAM microbiota compared to the luminal counterpart. These genera are fermenting bacteria able to decrease local pH by organic acids production, promoting host mucosal tissues protection from invading microorganisms (Van den Abbeele et al., 2011).

On the other hand, LAM microbiota showed a higher biodiversity and an enrichment in *Bacteroides, Prevotella*, and *Oscillospira*, genera able to digest and degrade biopolymers in the large intestine, and, in particular, polysaccharides, having enzymes that can target and degrade resistant dietary polymers, such as plant cell wall compounds (e.g., cellulose, pectin, and xylan) (Thomas et al., 2011).

The timing of mucosal and stool sample collection, 3 weeks apart, might represent a limitation of the study. However, Jalanka et al. (2015) observed that after a two-dose bowel preparation with MoviPrep colon cleansing, fecal microbiota before cleansing and after 14 and 30 days are comparable in microbial profiles.

Functional inference analysis by PICRUSt showed that LAM is enriched in genes encoding enzymes mostly involved in energy extraction from carbohydrates and lipids, whereas MAM is characterized by an increase in enzymes implicated in metabolism of amino acids and vitamins.

Microbiota composition can be affected by both host and environmental factors (Statovci et al., 2017); among the latter, diet is undoubtedly one of the major players in shaping our gut microbial community and microbial metabolite production (Thomas et al., 2011). Considering this and some recent data on the influence of total body fat content on gut microbiota (Haro et al., 2016; Statovci et al., 2017), we enrolled subjects with BMI in the normal and in the obese range to investigate several aspects that could influence the two niches, LAM and MAM.

We did not observe significant differences in dietary intake, collected by self-reported food daily diary, within NW and obese subjects. Literature data depict the phenomenon of the influence of psychological factors (guilt, insecurity, low self-esteem) in food-intake self-reporting by obese subjects (Hill and Davies, 2001). With the aim to avoid this bias, we combined the food diary with a questionnaire about food habits, physical activity and lifestyle. This questionnaire provides a more exhaustive tool for the assessment of dietary behavior in obese subjects (Turconi et al., 2008). Indeed, we observed a discrepancy between the self-reported diet and lifestyle habits in the obese group, with a reported balanced diet that contrasts with unhealthy behavior and anthropometric data. Biochemical analyses corroborate this finding, revealing for subjects with BMI > 30 a dyslipidemia with elevated triglycerides and LDL, and low HDL. Insulin, glycated hemoglobin and insulin resistance index were also increased in the obese group, showing an impaired insulin sensitivity and glucose tolerance, as previously reported in literature (Zeyda and Stulnig, 2009).

We found LAM community to be influenced by BMI status, in agreement with previous studies describing BMI as a good biomarker for bacteria dysbiosis (Riva et al., 2016; Borgo et al., 2017; Yun et al., 2017). In particular, we observed a significant increase in *Veillonellaceae*, known propionate and acetate producers (Lecomte et al., 2015), in obese subjects, whereas NW individuals were enriched in *Oscillospira* genus, belonging to the butyrate-producer family *Ruminococcaceae*.

Butyrate is the preferred energy source for colonic epithelial cells (Bergman, 1990) and promotes the reinforcement of intestinal epithelial barrier integrity (Peng et al., 2007). *Oscillospira* spp. has previously been associated with a vegetable-rich diet such as the Mediterranean diet (Konikoff and Gophna, 2016), reinforcing the key role of diet in shaping a healthy microbiota.

To understand whether specific bacterial taxa could be considered predictive markers of obesity/healthy status, we performed the indicator species analysis. As confirmed by other authors (Hippe et al., 2013; Tilg and Moschen, 2014; Kasai et al., 2016), the butyrate producers *Faecalibacterium prausnitzii* and *Flavonifractor plautii* are depleted in obese subject microbiota. Indeed, butyrate exerts a profound immune-metabolic effect, playing a key role in regulating metabolic inflammation (Hippe et al., 2013). Our data suggested that *Faecalibacterium prausnitzii* and *Flavonifractor plautii* could be considered as a crucial species for a healthy gut.

In light of recent findings by Sze and Schloss (2016), suggesting the existence of sample size effect on microbiota observations in obesity, further studies on huger cohorts are needed to corroborate a reduction of the above-mentioned genera.

In contrast with LAM data, analyses performed on MAM microbial population have shown a significantly grouping according to sex, as already reported by other authors that showed sex bias in both human and animal gut microbial communities (Mueller et al., 2006; Li et al., 2008; Ahonen et al., 2012; Dominianni et al., 2015). In particular, female MAM was enriched in *Actinobacteria* phylum (with an increase trend of the genus *Bifidobacterium*), and a significantly depletion in *Veillonellaceae*. Interestingly, we found the species *G. formicilis* associated with male and *B. adolescentis* with female MAM samples, an association that, to our knowledge, was never reported before. Org et al. (2016) demonstrated a relationship between sex hormones and gut microbiota. Their data suggest a role of sex-related factors in modulating gut bacterial communities in a rodent animal model, but the mechanism of this interplay is still unknown.

Future studies in humans are needed to better understand the relationship between microbiota and sex that in turn could identify novel factors for improving diagnostic and clinical strategies.

## Conclusion

Our findings corroborate previous studies showing the complex microbial geography within the gut, and point out that fecal sample alone could be not exhaustive for depicting gut microbiota. Indeed, the two niches could be affected by diverse host factors. The observed sex-related microbial signature, in particular, is still a quite unexplored field with possible important consequences in gender-specific medicine.

## Author Contributions

FB, EB, and AP designed the study, performed experiments and data analysis, and drafted the manuscript. AR and MS performed microbiota data analysis. SG and AB performed subject enrollment and analyzed clinical data. CL and GM performed supervision and writing – review and editing.

## Conflict of Interest Statement

The authors declare that the research was conducted in the absence of any commercial or financial relationships that could be construed as a potential conflict of interest.

## Footnotes

1 http://www.bda-ieo.it

2 http://greengenes.lbl.gov

3 https://www.r-project.org/

4 https://blast.ncbi.nlm.nih.gov/Blast.cgi

5 https://huttenhower.sph.harvard.edu/galaxy/root
