# Supplementary material for: Body Mass Index and Sex Affect Diverse Microbial Niches within the Gut
Source: Front Microbiol. 2018 Feb 14;9:213. doi: 10.3389/fmicb.2018.00213 (PMC5817072; doi:10.3389/fmicb.2018.00213)
Supplement: Supplementary file 1 [file Table_1.docx]

Table S1. Relative abundance at different taxonomic levels in LAM samples.

|  | **Taxa** | **O** (mean ± sd) | **NW** (mean ± sd) |
| --- | --- | --- | --- |
| *Phylum* | *Firmicutes* | 53.8±11.3 | 51.2±8.1 |
|  | *Bacteroidetes* | 38.5±12.7 | 41.7±7.9 |
|  | *Proteobacteria* | 3.5±2.7 | 4.5±5.7 |
|  | *Actinobacteria* | 1.5±1.2 | 1.4±1.9 |
|  | *Verrucomicrobia* | 2.4±5.3 | 0.9±1.7 |
| *Family* | *Ruminococcaceae* | 25.6±9.1 | 28.4±5.9 |
|  | *Lachnospiraceae* | 14.3±7.5 | 13.6±5.4 |
|  | *Bacteroidaceae* | 20±13.4 | 22.1±8.7 |
|  | *Veillonellaceae* | 9.0±4.8** | 4.2±2.0 |
|  | *Erysipelotrichaceae* | 0.3±0.1 | 0.3±0.2 |
|  | *Enterobacteriaceae* | 1.3±2.3 | 1.9±5.6 |
|  | *Streptococcaceae* | 0.7±1.4 | 0.2±0.2 |
|  | *Prevotellaceae* | 8.0±12.4 | 4.7±6.9 |
|  | *Bifidobacteriaceae* | 1.1±1.1 | 1.0±1.7 |
|  | *Rikenellaceae* | 4.6±3.2 | 5.9±3.8 |
|  | *Verrucomicrobiaceae* | 2.4±5.3 | 0.9±1.7 |
|  | *Porphyromonadaceae* | 2.0±1.4 | 2.9±2.0 |
|  | *Desulfovibrionaceae* | 0.6±0.6 | 0.9±0.7 |
|  | *Clostridiaceae* | 0.2±0.1 | 0.2±0.1 |
|  | *Alcaligenaceae* | 1.0±1.4 | 1.2±1.9 |
| *Genus* | *Bacteroides* | 20±13.4 | 22.1±8.7 |
|  | *Faecalibacterium* | 7.6±4.5 | 7.2±3.5 |
|  | *Streptococcus* | 0.7±1.4 | 0.2±0.2 |
|  | *Prevotella* | 8.0±12.4 | 4.6±6.8 |
|  | *Oscillospira* | 4.9±3.7 | 6.9±2.3* |
|  | *Bifidobacterium* | 1.1±1.1 | 1.0±1.7 |
|  | *Roseburia* | 4.7±5.9 | 3.4±2.2 |
|  | *Phascolarctobacterium* | 2.3±2.5 | 2.2±1.5 |
|  | *Ruminococcus* | 2.4±2.2 | 3.3±2.8 |
|  | *Parabacteroides* | 2.0±1.4 | 2.9±1.9 |
|  | *Coprococcus* | 1.2±1.6 | 1.9±2.0 |
|  | *Desulfovibrio* | 0.4±0.6 | 0.5±0.7 |
|  | *Dialister* | 3.6±4.2* | 0.8±1.4 |

Data are expressed as mean ± standard deviation. O, obese; NW, normal weight.

Reference range for biochemical parameters. Statistical significant values (*p ≤ 0.05; **p<0.001; *** p<0.0001) are reported highlighting the group with greater abundance.
