# Supplementary material for: Body Mass Index and Sex Affect Diverse Microbial Niches within the Gut
Source: Front Microbiol. 2018 Feb 14;9:213. doi: 10.3389/fmicb.2018.00213 (PMC5817072; doi:10.3389/fmicb.2018.00213)
Supplement: Supplementary file 2 [file Table_2.docx]

Table S2. Relative abundance at different taxonomic levels in MAM samples.

|  | **Taxa** | **M** (mean ± sd) | **F** (mean ± sd) |
| --- | --- | --- | --- |
| *Phylum* | *Firmicutes* | 65.4±16.2 | 59.0±17.3 |
|  | *Bacteroidetes* | 1.0±1.3 | 1.7±2.4 |
|  | *Proteobacteria* | 18.8±18.2 | 19.3±18.8 |
|  | *Actinobacteria* | 6.5±4.7 | 13.1±14.2* |
| *Family* | *Verrucomicrobia* | 0.7±1.5 | 0.9±1.6 |
|  | *Ruminococcaceae* | 5.4±4.2 | 8.1±6.6 |
|  | *Lachnospiraceae* | 7.7±10.5 | 10.2±12 |
|  | *Bacteroidaceae* | 0.2±0.5 | 0.3±0.3 |
|  | *Veillonellaceae* | 10.6±12.5* | 3.5±4.1 |
|  | *Erysipelotrichaceae* | 14.2±16.7 | 13.9±14.3 |
|  | *Enterobacteriaceae* | 8.4±19.2 | 5.8±7.4 |
|  | *Streptococcaceae* | 4.1±3.4 | 8.2±7.5 |
|  | *Prevotellaceae* | 0.2±0.3 | 0.5±1.8 |
|  | *Bifidobacteriaceae* | 1.0±1.3 | 6.4±12.6 |
|  | *Peptostreptococcaceae* | 4.7±8.2 | 3.8±5.6 |
|  | *Pasteurellaceae* | 2.1±5.4 | 5.3±10.4 |
|  | *Coriobacteriaceae* | 2.4±3.3 | 3.3±2.7 |
|  | *Comamonadaceae* | 4.4±5.7 | 2.1±2.3 |
|  | *Verrucomicrobiaceae* | 1.9±4.4 | 1.5±3.0 |
|  | *Desulfovibrionaceae* | 0.9±1.1 | 2.7±6.5 |
|  | *Clostridiaceae* | 0.6±1.6 | 0.1±0.1 |
| *Genus* | *Bacteroides* | 0.2±0.5 | 0.3±0.3 |
|  | *Faecalibacterium* | 3.1±2.8 | 5.8±5.7 |
|  | *Streptococcus* | 4.1±3.4 | 8.2±7.5 |
|  | *Clostridium* | 3.9±14.8 | 1.4±2.8 |
|  | *Prevotella* | 0.2±0.3 | 0.5±1.8 |
|  | *Bifidobacterium* | 1.0±1.2 | 6.2±12.5 |
|  | *Shigella* | 4.9±10.3 | 5.6±7.5 |
|  | *Enterobacter* | 2.9±8.2 | 0.2±0.3 |
|  | *Ruminococcus* | 0.1±0.2 | 0.1±0.2 |
|  | *Pasteurella* | 1.4±3.2 | 4.2±8.0 |
|  | *Parabacteroides* | 0.1±0.4 | 0.5±1.3 |
|  | *Desulfovibrio* | 0.9±1.1 | 2.7±6.5 |
|  | *Coriobacterium* | 1.0±1.0 | 1.4±1.5 |
|  | *Eubacterium* | 2.3±8.3 | 1.0±3.0 |
|  | *Unclassified clostridia* | 1.2±2.2 | 0.3±0.3 |

Data are expressed as mean ± standard deviation. M, male; F, female.

Statistical significant values (*p ≤ 0.05) are reported highlighting the group with greater abundance.
