# Supplementary figures and images for: Body Mass Index and Sex Affect Diverse Microbial Niches within the Gut
Source: Front Microbiol. 2018 Feb 14;9:213. doi: 10.3389/fmicb.2018.00213 (PMC5817072; doi:10.3389/fmicb.2018.00213)

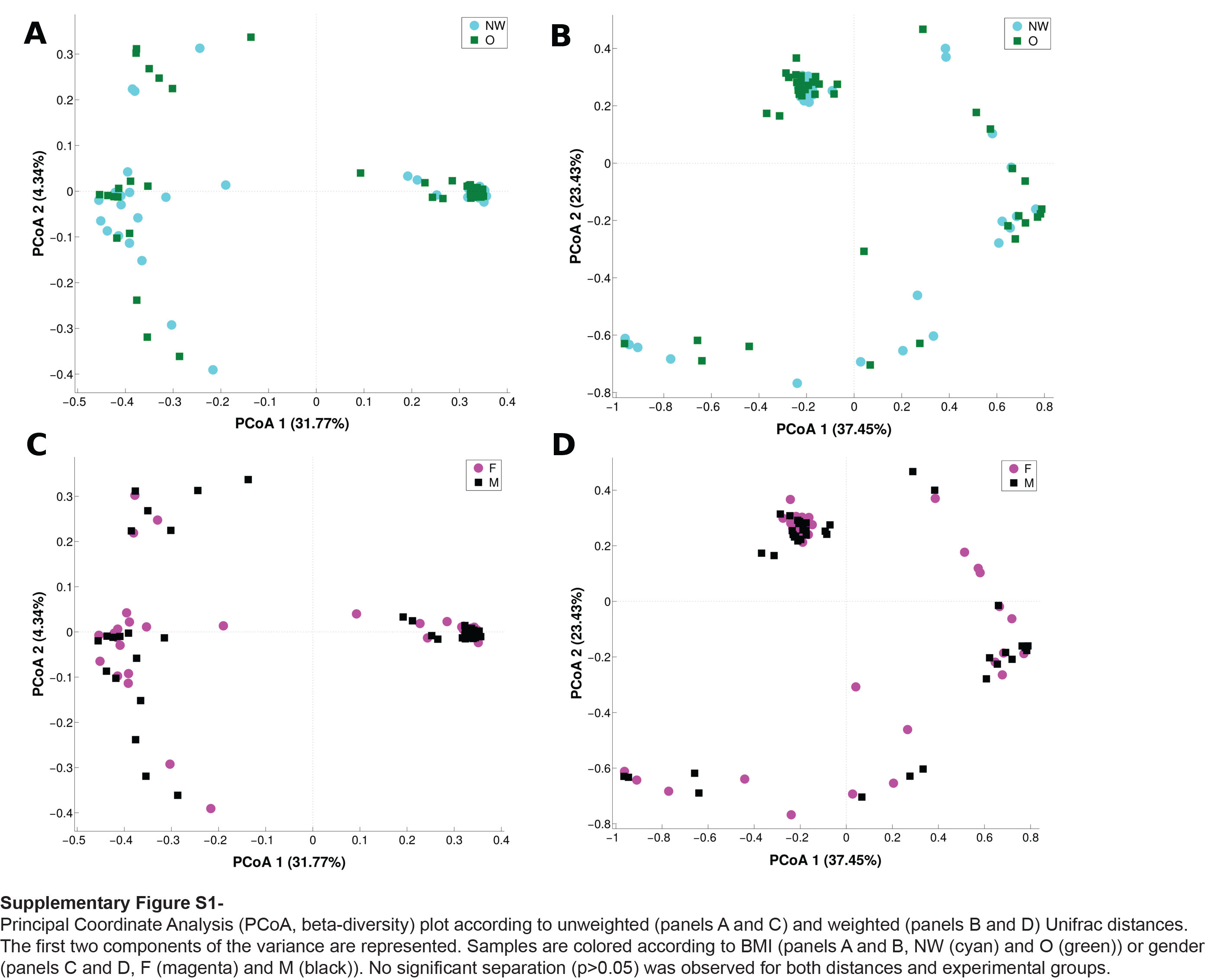

Supplement: Supplementary file 3 [file Image_1.TIF]
